# Mitochondrial Impairment: A Link for Inflammatory Responses Activation in the Cardiorenal Syndrome Type 4

**DOI:** 10.3390/ijms242115875

**Published:** 2023-11-01

**Authors:** Isabel Amador-Martínez, Omar Emiliano Aparicio-Trejo, Bismarck Bernabe-Yepes, Ana Karina Aranda-Rivera, Alfredo Cruz-Gregorio, Laura Gabriela Sánchez-Lozada, José Pedraza-Chaverri, Edilia Tapia

**Affiliations:** 1 Posgrado en Ciencias Biológicas, Universidad Nacional Autónoma de México, Ciudad Universitaria, Mexico City 04510, Mexico; amador_i@ciencias.unam.mx (I.A.-M.); anitaaranda023@comunidad.unam.mx (A.K.A.-R.); 2 Departamento de Fisiopatología Cardio-Renal, Instituto Nacional de Cardiología Ignacio Chávez, Mexico City 14080, Mexico; omar.aparicio@cardiologia.org.mx (O.E.A.-T.); laura.sanchez@cardiologia.org.mx (L.G.S.-L.); 3 Departamento de Biomedicina Cardiovascular, Instituto Nacional de Cardiología Ignacio Chávez, Mexico City 14080, Mexico; bis_by@comunidad.unam.mx; 4 Laboratorio F-315, Departamento de Biología, Facultad de Química, Universidad Nacional Autónoma de México, Mexico City 04510, Mexico; pedraza@unam.mx; 5 Departamento de Fisiología, Instituto Nacional de Cardiología Ignacio Chávez, Mexico City 14080, Mexico; alfredo.cruz@cardiologia.org.mx

**Keywords:** chronic kidney disease, cardiorenal syndrome type 4, mitochondria, innate immune response, chemokines, inflammation

## Abstract

Cardiorenal syndrome type 4 (CRS type 4) occurs when chronic kidney disease (CKD) leads to cardiovascular damage, resulting in high morbidity and mortality rates. Mitochondria, vital organelles responsible for essential cellular functions, can become dysfunctional in CKD. This dysfunction can trigger inflammatory responses in distant organs by releasing Damage-associated molecular patterns (DAMPs). These DAMPs are recognized by immune receptors within cells, including Toll-like receptors (TLR) like TLR2, TLR4, and TLR9, the nucleotide-binding domain, leucine-rich-containing family pyrin domain-containing-3 (NLRP3) inflammasome, and the cyclic guanosine monophosphate (cGMP)–adenosine monophosphate (AMP) synthase (cGAS)–stimulator of interferon genes (cGAS-STING) pathway. Activation of these immune receptors leads to the increased expression of cytokines and chemokines. Excessive chemokine stimulation results in the recruitment of inflammatory cells into tissues, causing chronic damage. Experimental studies have demonstrated that chemokines are upregulated in the heart during CKD, contributing to CRS type 4. Conversely, chemokine inhibitors have been shown to reduce chronic inflammation and prevent cardiorenal impairment. However, the molecular connection between mitochondrial DAMPs and inflammatory pathways responsible for chemokine overactivation in CRS type 4 has not been explored. In this review, we delve into mechanistic insights and discuss how various mitochondrial DAMPs released by the kidney during CKD can activate TLRs, NLRP3, and cGAS-STING immune pathways in the heart. This activation leads to the upregulation of chemokines, ultimately culminating in the establishment of CRS type 4. Furthermore, we propose using chemokine inhibitors as potential strategies for preventing CRS type 4.

## 1. Cardiorenal Syndrome Overview

The intrinsic association between cardiovascular disease (CVD) and kidney disease was first described by Robert Bright over a century ago [1]. This maladaptive link is termed cardiorenal syndrome (CRS) [2]. CRS comprises five distinct subtypes classified based on the initial pathology and temporality, as detailed in Table 1. Each subtype of CRS prevails due to the high global prevalence of both cardiac and renal diseases [3,4,5,6,7]. Chronic kidney disease (CKD), for instance, affects approximately 10–12% of the world’s population [6,8] and ranks as the 12th leading cause of death. This predicament is projected to escalate due to aging, diabetes, and hypertension [7]. CKD is characterized by persistent structural or functional renal alterations, often defined by a glomerular filtration rate (GFR) of less than 60 mL/min/1.73 m^2^ [9,10].

CKD substantially augments the risk of cardiovascular mortality, with CKD patients facing a 10–30 fold higher risk of cardiac-related death, a risk that escalates with declining GFR [19,20,21]. The primary cardiovascular complications and causes of mortality associated with CKD encompass ischemic heart disease, peripheral vascular disease, stroke, and heart failure [7,22,23,24]. In the context of CRS classification, CRS type 4 emerges when CKD plays a contributory role in CVD (as outlined in Table 1) [11,25]. CRS type 4 poses a significant public health concern, yet its underlying pathophysiology remains inadequately understood [17]. Furthermore, there is an alarming absence of effective therapies to mitigate the proliferation of cardiovascular complications during CKD, and patients often face early mortality despite undergoing renal replacement therapies [17,18,26]. As such, the development of urgent treatments and the identification of potential intervention targets are imperative.

### The Kidney-Heart Crosstalk

The causes of CRS type 4 are multifactorial and encompass a range of factors, including hemodynamic alterations, dysregulation of neurohormonal responses, overactivation of the renin–angiotensin–aldosterone system (RAAS), anemia, and pressure overload, among others [11,17,27]. Additionally, as renal function declines, the accumulation of uremic toxins becomes a significant concern [28,29]. These factors have garnered attention for their roles in inducing cardiovascular alterations secondary to CKD.

Consequently, uremic patients often experience endothelial dysfunction, a prevalent complication of CKD [30,31]. Moreover, kidney–heart crosstalk is mediated by the systemic trafficking of extracellular vesicles (EV). This concept gains support from discovering EV-containing proteins in cardiac tissue that are not typically found in the heart but show increased presence in the kidney [32]. Such interorgan communication can shed light on heart dysregulation in the context of CKD.

In response to proinflammatory insults, proximal tubule cells release exosomes, a specific type of EV, which carry dysregulated micro ribonucleic acids (RNAs) associated with the regulation of proinflammatory and profibrotic pathways, as well as dysregulated mitochondrial RNAs [33]. As CKD progresses, uremic toxins continue to accumulate, exacerbating inflammation and oxidative stress in the kidney [28,34]. This accumulation likely induces inflammasome activation and pyroptosis by releasing cellular and mitochondrial components [35]. Notably, mitochondrial components are identified as mitochondrial damage-associated molecular patterns (mtDAMPs), and they trigger inflammatory and immune responses, contributing to inflammation in various organs [36,37,38].

Mitochondrial DAMPs stimulate innate immune signaling responses in different cardiac cell lineages, triggering the activation of transcription factors such as the nuclear factor-kappa B (NF-κB), a crucial factor for inflammation [39,40]. In addition, mitochondria provide an assembly platform for signaling innate immune responses, contributing to additional inflammatory responses [41]. The main innate immune responses that are activated during the cardiorenal association include Toll-like receptors (TLRs), the nucleotide-binding domain-like receptor family pyrin domain-containing protein 3 (NLRP3) inflammasome, and cyclic guanosine monophosphate (cGMP)–adenosine monophosphate (AMP) synthase (cGAS)–stimulator of interferon genes (STING) [42,43,44,45,46]. TLRs, NLRP3, and cGAS-STING pathways produce the upregulation of cytokines, vasoactive substances, chemokines, and inflammatory responses [46,47,48]. Chemokine’s overstimulation produces the recruitment of leukocytes to tissues and dysregulated infiltration, leading to chronic cardiac damage [49,50,51]. Interestingly, experimental studies have shown that chemokines are upregulated in the heart during CKD, establishing a link for CRS type 4 development [52,53].

Chemokine inhibitors have shown promise in reducing chronic inflammation and preventing cardiac and cardiorenal impairment [52,53,54,55]. Despite these advancements, the molecular mechanisms underlying how mtDAMPs are released by the kidneys in CKD may trigger innate immune pathways in the heart, ultimately leading to chemokine overactivation and the development of CRS type 4, remain poorly understood.

This review aims to provide a comprehensive understanding of the molecular mechanisms involved in mtDAMP release within the context of CKD. Special attention will be given to the role of kidney-derived mtDAMPs in initiating innate immune pathways such as TLRs, NLRP3 inflammasome, and cGAS-STING pathway in the heart.

Additionally, we explore the identification of chemokines associated with the cardiorenal connection. Finally, we will review the potential use of chemokine inhibitors as a strategy for preventing CRS type 4. By addressing these aspects, this review aims to shed light on the intricate interplay between CKD, mtDAMPs, chemokines, and CRS type 4, contributing to our understanding of these complex medical conditions.

## 2. Mitochondrial Dysfunction and Inflammatory Alterations in CRS Type 4

**Figure 1:**
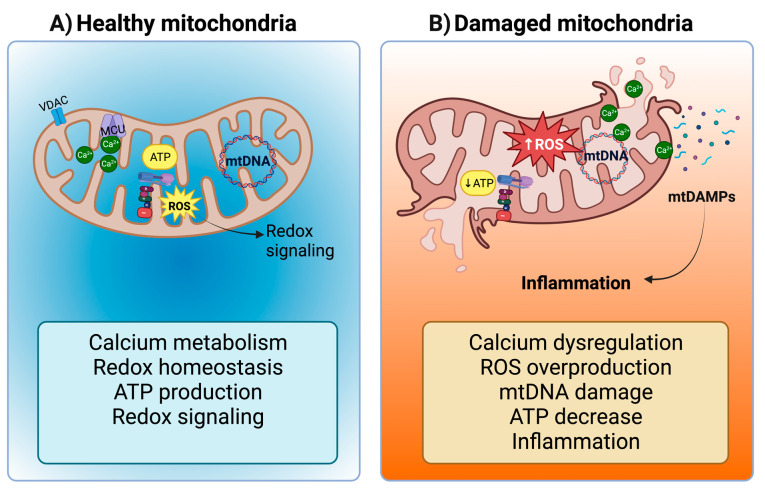
Mitochondria in tubular cells and cardiomyocytes. (**A**) Under normal conditions, mitochondria regulate calcium (Ca^2+^) metabolism (Ca^2+^ ions pass the mitochondrial outer membrane (MOM) through the Voltage-dependent Anion Channel (VDAC) and the mitochondrial inner membrane (MIM) through the mitochondrial calcium uniporter (MCU)), redox homeostasis, adenosine triphosphate (ATP) production, and reactive oxygen species (ROS) that act as second messengers for redox signaling. However, (**B**) mitochondrial damage leads to Ca^2+^ dysregulation, ROS overproduction, mitochondrial deoxyribonucleic acid (mtDNA) damage, ATP decrease, and finally, inflammation, the latter being induced by the release of mitochondrial damage-associated molecular patterns (mtDAMPs). Figure created by using Biorender.com.

Mitochondria are versatile organelles with diverse roles encompassing biosynthesis, metabolism, calcium (Ca^2+^) regulation, inflammation, and cell death, among other crucial cellular processes [56]. Importantly, mitochondria are also the primary source of reactive oxygen species (ROS), particularly in complexes I and III of the electron transport system (ETS) [57]. At moderate levels, these ROS function as secondary messengers, governing intracellular signal transduction cascades (Figure 1A) [58,59,60]. However, excess ROS production, often associated with reduced oxidative phosphorylation and ETS activity, leads to oxidative stress (Figure 1B). Notably, due to their high energy demands, the heart and kidneys have a dense population of mitochondria [58,61,62,63]. Consequently, mitochondrial dysfunction serves as a potent trigger for the development of renal, cardiac, and cardiorenal diseases.

Another critical characteristic of mitochondria is their ability to promptly detect and respond to insults through morphological changes, bioenergetic adaptations, self-renewal, and degradation [63,64]. However, in the context of CKD, fluid and electrolyte imbalance, retention of fluids, increased blood pressure, ROS overproduction, and hypertrophy instigate a cascade of mitochondria alterations, beginning in proximal tubular epithelial cells and subsequently affecting cardiomyocytes [63,65]. Furthermore, these maladaptive responses in tissues lead to functional declines in renal and cardiac mitochondria, culminating in inflammatory processes [62,66]. These responses are initiated by mitochondrial components typically located in the extracellular space or peripheral circulation derived from damaged renal cells [67,68]. Elevated levels of circulating mitochondrial components may inflict damage on the mitochondria of distant organs, disrupting ROS balance and provoking inflammation [41].

In the initial stages of inflammation, immune cells like neutrophils are recruited to phagocytose and clear dead cells and matrix debris, facilitating inflammation resolution despite the concomitant generation of ROS and inflammatory mediators [69,70,71]. Additionally, disturbances in anti-inflammatory processes interconnect the innate and adaptive immune systems, promoting oxidative stress and apoptosis in renal and cardiac cells [72,73]. Consequently, mitochondrial alterations and inflammatory responses are intrinsic mechanisms in establishing CRS type 4.

### 2.1. Mitochondrial Dysfunction in CKD Activates the NLRP3-NF-κB Pathway

In severe stages of CKD or during hemodialysis, elevated levels of the NLRP3 inflammasome and its activators, including uremic toxins, oxidative stress, and mitochondrial deoxyribonucleic acid (mtDNA), are observed in the serum or urine. These findings underscore the persistence of inflammation and fibrosis [74,75,76]. Inflammasomes are cytoplasmic multi-protein signaling complexes that mediate the host’s immune response to cellular damage and infection [77]. When NLRP3 is exposed to pattern-associated molecular patterns (PAMPs) such as viruses or bacteria, or DAMPs, it undergoes release and oligomerization through its central nucleotide-binding (NACHT) domain [78]. The NLRP3 inflammasome triggers the activation of the transcription factor NF-κB [79,80,81], thereby initiating additional inflammatory responses.

The NF-κB family encompasses distinct but related transcription factors, including p50, p52, p65 (RelA), c-Rel, and RelB [81,82]. These components form dimers and bind to specific DNA target sequences known as “κB” sites to modulate gene expression [79,81]. Among the many target genes under NF-κB’s control are cytokines (tumor necrosis factor-alpha (TNF-α), interleukins (IL-) IL-1β, IL-6, and IL-12), adhesion molecules, and some chemokines (CCL2, IL-18, CCL5, CXCL2, CXCL1, and CXCL10) [83]. Importantly, the orchestration of NF-κB activation involves a critical role played by the NLRP3 inflammasome and ROS, which promote the phosphorylation of the p65 subunit, thereby activating NF-κB [84].

Regardless of the underlying cause of CKD, renal mitochondria face challenges in meeting the increased demand for ATP [85,86]. This imbalance favors inorganic phosphate accumulation and increases oxygen uptake [87,88]. Notably, the reduction in adenosine triphosphate (ATP) production is consistently associated with lower oxidative phosphorylation (OXPHOS) due to decreased levels and/or activity of mitochondrial ETS complexes [89,90]. This decline in ETS activity, particularly in complexes I and III, augments mitochondrial ROS production and oxidative stress within these organelles [91,92]. Furthermore, the inhibition of complexes I and III activity is observed in the advanced stages of CKD [93] and can be induced by the activation of the NLRP3-NF-κB pathway [94]. Inhibition of complex I enhance the NLRP3 pathway by increasing ROS levels, promoting thiol oxidation [95]. In addition to the ETS inhibition, the Krebs cycle also exhibits dysfunction in CKD, as evidenced by decreased mRNA levels and reduced urinary Krebs cycle metabolites concentrations in patient renal biopsies [88,96].

In contrast, the renal succinate and fumarate levels increase in the unilateral ureteral renal obstruction (UUO) and chronic hypoxia CKD models, increasing ROS production [97,98]. Interestingly, the succinate accumulation induced by the hypoxia-inducible factor-1 alpha (HIF-1α) triggers macrophage stimulation, mediated by NLRP3 pathway activation, resulting in the rise of IL-1β and IL-18 secretion to the medium [99]. Furthermore, succinate dehydrogenase (complex II) inhibition by itaconic acid can reduce the NLRP3 activation and the release of pro-inflammatory molecules [100], suggesting a robust metabolic regulation of this inflammatory pathway. For example, in the diabetic kidney, renal succinate accumulation has been shown to reduce mitochondrial fatty acid β-oxidation [101], inducing the acetyl-coenzyme A (acetyl-CoA)/CoA ratio to increase. The acetyl-CoA excess can be excreted as acetylcarnitine in the urine. Remarkably, CKD patients show significantly increased serum acetylcarnitine concentrations, which correlate with the reduction of renal function [101].

In CKD, acetyl-CoA increase is associated with lipid intermediates and lipid derivatives accumulation in the kidney, leading to lipotoxicity [102,103]. Since early CKD stages, nephron segments like proximal tubules increase their lipid levels [96,102] and the fatty acid uptake protein CD36 levels [102,104], associated with inflammatory pathways induction [105]. CKD lipotoxicity increases fatty acid levels in plasma and kidneys, especially palmitic acid [103,106,107]. Palmitic acid has been shown to inhibit the adenosine monophosphate (AMP)-activated protein kinase (AMPK) signaling, reducing mitochondrial function, and increasing mitochondrial ROS production to favor the NLRP3 inflammasome activation, caspase-1, IL-1β, and IL-18 production [107,108]. This agrees with recent works suggesting that the early mitochondrial ETS alteration triggers the decrease in β-oxidation [89,93,109]. Advanced stages of CKD are also characterized by the downregulation of β-oxidation enzymes in the kidney [103,110,111], which induce inflammation in proximal tubules and glomeruli [109,112]. Interestingly, we previously showed in folic acid and 5/6 nephrectomy-induced CKD models that the reduction in palmitic acid β-oxidation is related to mitochondrial hydrogen peroxide production, coupling reduction, and mitochondrial fission induction, increasing inflammatory markers in the kidney [93,109]. The higher fatty acid synthesis in CKD may also induce NLRP3 pathway activation by the fatty acid synthase (FASN) induction. This enzyme is upregulated in the remnant kidney of 5/6 nephrectomy rats [106], likely leading to upregulation of the expression of the inflammasome components: NLRP3, caspase-1, and pro-IL-1β [99]. Thus, renal mitochondria impairment could be an essential regulator of NLRP3 inflammasome activation during CKD.

### 2.2. The Release of mtDAMPs during CKD and the Establishment of CRS Type 4

In the context of CKD, inflammation follows a distinct trajectory. Initially, it is characterized by functional alterations, including glomerular hyperfiltration, which serves as a compensatory mechanism [113,114]. However, structural changes emerge as time progresses, giving rise to a cascade of complications. These structural transformations encompass proteinuria, interstitial nephritis, tubular epithelial–mesenchymal transition, nephron fibrosis, and scarring [65,115,116].

The structural changes are attributed to elevated circulating levels of various molecules, including fibrinogen, TNF-α, and IL-1β and IL-6. These molecules trigger an inflammatory response and promote the secretion of fibrotic mediators [117,118]. Consequently, inflammation leads to a reduction in mitochondrial ATP production, mitochondrial uncoupling, and an increase in ROS, culminating in oxidative stress and mitochondrial damage [119,120].

In response to mitochondrial uncoupling or oxidative stress, cells activate apoptosis, a programmed type of cell death. Apoptosis entails the release of mitochondrial proteins to the cytosol. This process is initiated by the oligomerization of effector Bcl2-family proteins such as B-cell lymphoma 2 (BCL-2)-associated X (BAX) and BCL-2 antagonist/killer 1 (BAK). These proteins form oligomers that promote the permeabilization of the mitochondrial outer membrane (MOM). As a result, mtDAMPs are released from both the intermembrane mitochondrial space and the matrix to the cytosol [121,122].

Another main form of cell death is necrosis, characterized by the plasma membrane’s rupture, which releases intracellular contents. The necrotic process is regulated, and it encompasses various types of necrosis, including ferroptosis, necroptosis, and pyroptosis. Each of these distinct mechanisms contributes significantly to various kidney diseases, either by directly affecting kidney cells or by recruiting immune cells and triggering inflammatory responses [123].

Moreover, the innate immune system plays a significant role in initiating acute inflammation in CRS. This inflammatory response is triggered by recognizing DAMPs or mtDAMPs [124]. DAMPs are endogenous molecules that are released from cells, either passively during cell death processes or actively through exocytosis [121,125]. These molecules include heat shock proteins (HSP) 90, 70, 60, and 27 [126], uremic toxins [120,127], extracellular ATP, nucleic acids, and oxidative products derived from ROS [48,125].

On the other hand, mtDAMPs released by apoptosis or necrosis encompass mtDNA, cytochrome c, ATP, N-formyl peptide, succinate, and cardiolipin [121]. DAMPs and mtDAMPs play a pivotal role in activating the innate immune system, primarily by interacting with plasmatic and intracellular pattern-recognition receptors (PRRs) encoded in the germline [128,129]. These essential PRR families encompass Toll-like receptors (TLRs), C-type lectin receptors (CLRs), RIG-I-like receptors (RLRs), NOD-like receptors (NLRs), and intracellular DNA sensors like cGAS [42,45,130].

In the context of CRS type 4 pathophysiology, the activation of TLRs, NLRs, and cGAS receptors leads to the upregulation of genes involved in inflammatory responses. These genes encode inflammatory cytokines and chemokines, thereby initiating innate immune responses (discussed below) [39,41,131].

DAMPs and mtDAMPs can also trigger endothelial dysfunction and affect cellular membranes and mitochondria of the heart cells, resulting in premature cell death in the form of apoptosis and necrosis [132]. In cardiac diseases, necrotic cell death further exacerbates the upregulation of proinflammatory cytokines and chemokines and the recruitment of inflammatory cells such as neutrophils, macrophages, and mast cells, thereby worsening inflammation [133]. The persistent inflammatory stimulus also leads to fibroblast activation and cell proliferation, leading to fibrosis [36,133,134]. These processes collectively contribute to chronic injuries and unremitting inflammation, establishing a positive feedback loop between mitochondrial alterations and the activation of immune pathways, ultimately culminating in the establishment of CRS type 4.

### 2.3. NLRP3-NF-κB Pathway Activation in the Heart by CKD-Derived mtDAMPs and ROS

One of the different important stimuli for ROS production and immune activation in CKD occurs following the dissociation of the thioredoxin (TRX) complex from the thioredoxin-interacting protein (TXNIP), ultimately leading to the activation of the NLRP3 inflammasome [135,136]. TRX are ubiquitously present redox-active proteins known for their antioxidant and anti-inflammatory properties. Elevated ROS levels disrupt the TRX complex, causing TXNIP to bind to the leucine-rich repeat region of NLRP3, consequently activating the inflammasome [137]. Notably, the administration of recombinant TRX has shown promise in ameliorating renal damage and preventing the progression of CKD [136].

In the context of CVD, several CKD-related alterations, including increased levels of angiotensin II levels, ROS production, reduced activity of antioxidant enzymes, and inflammation, contribute to decreased levels of thioredoxins [138,139,140]. Furthermore, the depletion of mitochondrial TRX2 in cardiomyocytes leads to hypertrophy and disrupts mitochondrial respiratory function by reducing AMPK activity [141]. Impaired mitochondrial function and the activation of the NLRP3 inflammasome are associated with decreased levels of TRX2 during myocardial ischemia–reperfusion injury [142]. Intriguingly, elevated levels of NLRP3 and IL-1β observed in patients with coronary artery disease exhibit an inverse association with the expression and protein levels of TXNIP and TRX1 [143]. Additionally, in a rat model of diabetes-associated aortic remodeling, the reduction of ROS levels improved TXNIP protein levels and prevented an increase in the mRNA levels of NF-κB, NLRP3, and IL-1β [144,145]. These findings underscore the critical role of ROS in inducing inflammatory activation through alterations in the TRX system.

The rise in ROS levels due to disturbances in redox balance can also lead to the oxidation of mtDNA, a mtDAMP that activates the NLRP3 inflammasome [146]. Additionally, ROS can directly activate NLRP3 inflammasome in CVD [147]. These ROS may especially damage cardiomyocytes by activating the NF-κB pathway and the NLRP3 inflammasome [148]. In addition, activation of the NLRP3-NF-κB-ROS pathway in CKD not only initiates inflammation but also triggers additional mechanisms contributing to cardiorenal disturbances. For instance, ROS levels may activate the transforming growth factor beta (TGFβ-1), a key player in cardiac fibrosis [149]. TGFβ-1, in turn, further elevates ROS levels, promoting the activation of intracellular Smad pathways, leading to fibrosis, and decreasing antioxidant enzyme levels [149,150]. The consequence of reduced levels of antioxidant enzyme levels following kidney injury is the promotion of oxidative stress. This increase in ROS, primarily produced by mitochondria and NADPH oxidases (NOXs) in the heart, sets the stage for a cascade of events. These events include hemodynamic alterations, dysregulation of neurohormonal responses, overproduction of angiotensin I, and pressure overload, among others [11,17,27].

On the other hand, TNF-α, a pro-inflammatory cytokine known to induce cardiac hypertrophy, fibrosis, dysfunction under pressure overload, and chronic heart injuries [151,152], may trigger the activation of NLRP3 through the elevation of ROS levels [153,154]. This phenomenon can be elucidated by the chronic exposure of cells to TNF-α, which sets off p38-mitogen-activated protein kinases (MAPK) signaling, instigates inflammatory phenotypes, and suppresses the expression of antioxidant genes, resulting in an increase in ROS levels [155].

**Figure 2:**
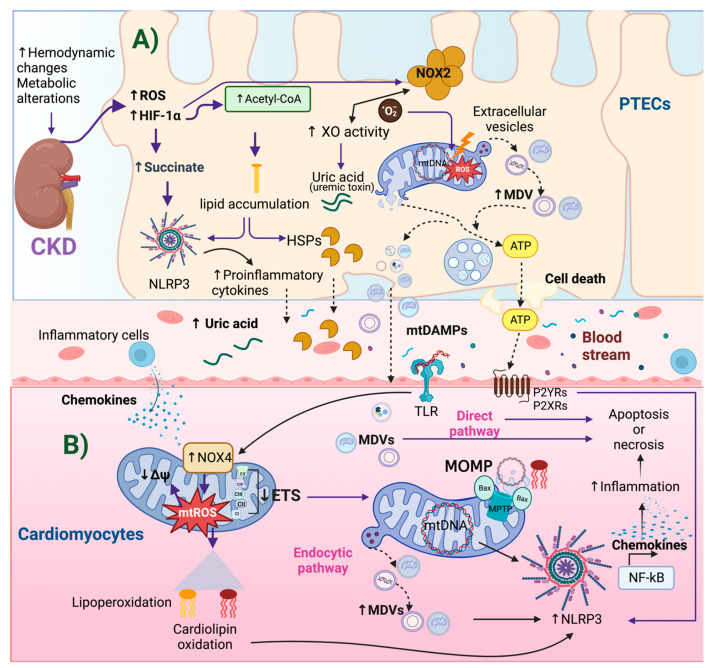
Mechanisms of mtDAMPs release in CKD and NLRP3 activation in cardiomyocytes during CRS type 4. (**A**) In chronic kidney disease (CKD), hemodynamic changes and metabolic alterations, mainly in proximal tubular epithelial cells (PTECs), cause reactive oxygen species (ROS) overproduction, activating hypoxia-inducible factor-1 alpha (HIF-1α). HIF-1α induces renal succinate accumulation, leading to β-oxidation decrease and acetyl-CoA and lipid accumulation. The increase in superoxide anion (·O_2_^−^) can be determined through NADPH oxidase 2 (NOX2), found in the cytosolic membrane, which causes damage to mitochondria and mitochondrial deoxyribonucleic acid DNA (mtDNA). The upregulation of xanthine oxidase (XO) activity in CKD also produces ·O_2_^−^ anion and, importantly, its last product is the uremic toxin: uric acid. ROS also induce the activation of heat shock proteins (HSPs) and, along with uric acid, participate as Damage-associated Molecular Patterns (DAMPs). Damaged mitochondria release their mitochondrial components in mitochondrial-derived vesicles (MDV) containing mtDNA, ATP, DNA, and mitochondrial fragments. (**B**) MDVs, HSPs, mtDAMPs, and uric acid are transported by blood. They might arrive at cardiomyocytes inducing inflammatory responses by pattern-recognition receptors such as Toll-like receptors (TLRs) or the nucleotide-binding domain-like receptor family pyrin domain-containing protein 3 (NLRP3). These receptors may produce the transcription of inflammatory genes, such as chemokines, which produce inflammatory cell infiltration into the heart cells. ATP can be recognized by purinergic receptors (P2YRs and P2XRs). One type is ionotropic receptors, such as P2XRs, that open upon the binding of ATP. This allows sodium and Ca^2+^ influx and potassium efflux. Potassium efflux activates the inflammasome in cardiomyocytes and inflammatory cells such as macrophages and dendritic cells. The release of mtDAMPs also damages cardiomyocytes’ mitochondria, which might be visualized by NOX4 overactivation, ROS increase, decreased electron transport system (ETS), loss of the mitochondrial membrane potential (ΔΨ), and cardiolipin oxidation. The damage in these mitochondria leads to cell death in the form of apoptosis or necrosis, promoting the exit of cardiomyocyte mitochondrial components via endocytic and direct pathways. This vicious cycle of mtDAMPs release and damage establishes CRS type 4. ATP: adenosine triphosphate, MOMP: mitochondria outer membrane permeabilization, MPTP: mitochondrial permeability transition pore opening, PTECs: proximal tubular epithelial cells, ↑: increase, ↓: decrease. Figure created by using BioRender.com.

Furthermore, the exposure of fibroblasts and human immune cells to TNF-α, in combination with oxidative stress, may prompt the degradation of the IκBα subunit by the IκB Kinase (IKK). This degradation event, in turn, leads to NF-κB activation and the transcription of genes associated with proinflammatory cytokines, chemokines, and NLRP3 inflammasome [156,157]. Interestingly, the antihyperglycemic peptide Nesfatin-1 has shown promise in ameliorating hypertrophy and heart dysfunction in uremic cardiomyopathy by mitigating ROS levels, suppressing p38-MAPK signaling, and reducing TNF-α levels [158]. In particular, the measurement of TNF-α levels in serum can serve as a predictor for cardiovascular mortality in CKD patients [159,160]. This underscores the significance of sustained inflammatory signaling and ROS during CKD in potentially contributing to the development of CRS type 4 (Figure 2).

### 2.4. Involvement of NLRP3 Inflammasome and Toll-like Receptors 2 and 4 in CRS Type 4

Overactivation of NLRP3 inflammasome has been linked to myocardial fibrosis, hypertrophy, and cardiac dysfunction [154,161]. The upregulation of NLRP3, IL-1β, and IL-18 in the heart during CKD is closely associated with exposure to DAMPs, thus highlighting the cardiorenal connection [146,162].

DAMPs and mtDAMPs can trigger NLRP3 activation through TLRs, including uremic toxins, mitochondrial components released due to defects in membrane integrity, mitochondrial ROS, and cardiolipin [45,131].

TLRs are type I integral transmembrane proteins composed of three main components: an ectodomain with leucine-reach repeats (LRRs), a transmembrane domain, and a cytoplasmic Toll/IL-1 receptor (TIR) domain. LRRs recognize PAMPs or DAMPs, while the TIR domain initiates the downstream signaling [128,163]. The primary functions of TLRs include stimulating phagocytosis and mediating inflammation by sensing molecules from damaged cells [164].

The activation of TLRs by ligands results in the dimerization of TLRs’ cytoplasmic signaling domains [165]. This TIR-TIR complex initiates downstream signaling by recruiting specific adaptor molecules [166]. DAMPs and mtDAMPs, such as debris from apoptotic and necrotic cells, inflammatory factors, nucleic acid fragments, oxidative products, and uremic toxins generated during renal damage can activate classical TLR2 and TLR4 pathways in the heart [51,71].

Upon recognition of DAMPs and mtDAMPs by TLR2 and TLR4, they stimulate macrophages to produce inflammatory cytokines [145,167,168]. TLR2 and TLR4 rely on adaptor molecules, with TLR2 engaging myeloid differentiation factor 88 (MyD88) and TLR4 utilizing Toll/IL-1 receptor (TIR) domain-containing adaptor inducing interferon beta (TRIF) [166]. Activation of the MyD88-dependent pathway involves the participation of IL-1 receptor-associated kinases (IRAK), including IRAK1 and IRAK4, TNF receptor-associated factor 6 (TRAF-6), and MAPK. These events culminate in the activation of the transcription factor NF-κB, leading to the production of proinflammatory cytokines such as pro-IL-1β and pro-IL-18 [71,169].

Sustained inflammation in the kidney may lead to the activation of TLR2 and TLR4 in the heart, thereby contributing to CRS type 4. Supporting this notion, the deletion of both TLRs during unilateral kidney ischemia/reperfusion has demonstrated a reduction in cardiac hypertrophy markers such as B-type natriuretic peptide and α-actin. This suggests that sustained inflammation in the kidney can upregulate TLRs in the heart [170]. Activation of the TLRs pathway induces the translocation of NF-κB to the nucleus, where it promotes transcription of inflammation mediators, including IFN-γ, IL-6, and TNF-α, all of which are associated with cardiac hypertrophy [171].

Another set of activators for TLR2 and TLR4 includes HSP proteins. HSPs are intracellular chaperones with a pivotal role in stress responses. In particular, HSP70 has been identified in the extracellular medium, where it is recognized as a DAMP and activates immune cells. This activation results in the secretion of pro-inflammatory cytokines such as IL-1β, IL-6, and TNF-α [172]. The release mechanism of extracellular HSP70 involves a membrane-associated form [173]. In the context of CKD, HSP70 levels appear elevated in the urine and serum of patients, which is closely associated with inflammation and immune responses [174,175]. Furthermore, high levels of HSP70 in plasma have been linked to cardiac alterations [176]. As such, systemic HSP70 during CKD may induce disturbances in the heart by activating TLR2 and TLR4.

It has been reported that the uremic toxin indoxyl sulfate induces an elevation in HSP90 levels, subsequently triggering the activation of TLR4. This suggests that HSP90 plays a pivotal role in promoting inflammation through the TLR4 pathway. Notably, HSP90 is a protein essential for sustaining renal vascular tone, as its inhibition has been associated with a reduction in GFR [177]. Recent studies have indicated that HSP90α, an isoform of HSP90, is required for its interaction with endothelial nitric oxide synthase (eNOS). However, in the context of CKD, this interaction is impaired, leading to a decreased availability of NO. This reduction in NO contributes to the development of CRS type 4 [178].

**Figure 3:**
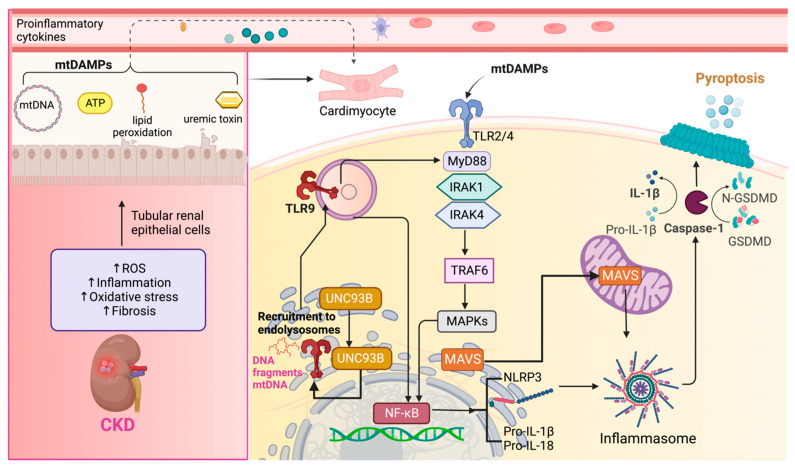
Toll-like receptors (TLRs) in cardiorenal syndrome type 4 (CRS type 4). In chronic kidney disease (CKD), several factors contribute to the initiation of inflammation, oxidative stress, and fibrosis in renal tubular epithelial cells. These cellular insults result in the release of Damage-associated Molecular Patterns (DAMPs), including mitochondrial deoxyribonucleic acid (mtDNA), ATP, peroxidized lipids, and uremic toxins, which play a pivotal role in mediating inflammatory responses in cardiomyocytes and the recruitment of inflammatory cells. mtDNA, extracellular ATP, peroxidized lipids, and uremic toxins can activate membrane-bound Toll-like receptors, specifically TLR2 and TLR4. The activation of these receptors is mediated by the adaptor molecule myeloid differentiation factor 88 (MyD88). Once TLR2 and TLR4 are engaged, they initiate the mitogen-activated protein kinases (MAPKs) pathway. Activated MAPKs ultimately lead to the activation of nuclear factor kappa B (NF-κB), a transcription factor involved in the regulation of inflammatory genes. NF-κB promotes the assembly of the NOD-like receptor (NLR) family pyrin domain containing 3 (NLRP3) inflammasome. Within this complex, pro-interleukin-1 beta (pro-IL-1β) and pro-interleukin-18 (pro-IL-18) are processed to form the mature and active IL-1β and IL-18. TLR9 might be activated by internal, external, or mtDNA, which induces the recruitment of TLR9 by UNC93B protein from the endoplasmic reticulum to endolysosomes. It leads to the activation of MAPKs mediated by MyD88 by following the same steps of TLR2 and TLR4. The activation of NLRP3 results in a cascade of events, ultimately leading to pyroptosis. Pyroptosis is mediated by gasdermin D (GSDMD), which forms pores in the cell membrane, causing cell lysis and the release of pro-inflammatory intracellular contents. The mitochondrial antiviral proteins (MAVS) also activate NLRP3, leading to pyroptosis induction. IL-1 receptor-associated kinases (IRAK), IRAK1 and IRAK4, TNF receptor-associated factor 6 (TRAF-6). Figure created by using Biorender.com.

Therefore, maintaining regular levels of HSP90 is necessary to prevent cardiovascular disturbances. However, it is important to note that an excessive increase in this protein could also potentially favor the development of CRS type 4. In summary, TLR2 and TLR4 activation and downstream inflammatory signaling are central factors contributing to cardiac disorders during CKD, ultimately promoting the establishment of CRS type 4 (Figure 3).

### 2.5. Role of TLR9 in Inflammation and Its Implication in CRS Type 4

Another TLR known to induce inflammation through the adaptor molecule MyD88 is TLR9 [166]. TLR9, primarily localized in endolysosomes, is associated with activating p38 MAPK signaling [179]. The inflammatory response initiated by TLR9 is triggered by DNA fragments rich in unmethylated cysteine–phosphate–guanine motifs, with mtDNA being a notable example [180]. These DNA fragments can be internalized in various tissues by dendritic cells and macrophages [162] and subsequently delivered to endolysosomes, where they activate TLR9 [166].

Upon activation, TLR9 interacts with the endoplasmic reticulum (ER) membrane protein UNC93B, facilitating its transportation to the endolysosomal compartment [181,182] (Figure 3). TLR9 activation leads to two distinct pathways [183]: one is associated with the transcriptional activation of proinflammatory cytokines, requiring the involvement of NF-κB, while the other relates to the activation of type I interferon genes [184].

In an experimental model of CKD induced by diabetic nephropathy, researchers observed an upregulation of TLR9 expression in the kidneys. This upregulation was linked to an increase in NF-κB activity and apoptosis, with a dependence on the upregulation of the NLRP3 inflammasome [185].

Activation of TLR9 increases in renal proximal tubular cells following ischemic injury, initiating a cascade of events that promote inflammation, apoptosis, and necrosis through NF-κB and caspase-dependent pathways [186]. Because of apoptosis or necrosis, renal DAMPs are released. These DAMPs may be exposed to the cell surface and released into the extracellular space, acting as potent inflammation triggers [125].

Renal DAMPs have the potential to activate TLR9 in cardiac cells, inducing oxidative stress and inflammatory responses, which can lead to the release of mtDNA or other mtDAMPs within cardiomyocytes. This process has been observed in mice cardiomyocytes, where the release of mtDNA after myocardial injury activated NF-κB through TLR9, ultimately contributing to cell death [187]. Furthermore, in patients with end-stage heart failure, the stimulation of TLR9 in cardiac tissue resulted in inflammation via the activation of NF-κB, causing additional cardiac damage [188]. The release of circulating mtDNA, potentially originating from the damaged kidney, could be responsible for this activation, especially in conditions like hypertension, where elevated levels of circulating mtDNA have been observed [189].

### 2.6. Extracellular Vesicles (EV) and Their Role in Inflammation

Exogenous mtDNA and other signaling molecules may also be transported into other cells, including cardiac cells, through extracellular vesicles (EVs). These EVs, such as exosomes, microvesicles, and apoptotic bodies, carry various cargo, including nucleic acids, proteins, and metabolic intermediaries [190,191].

Apoptotic bodies, in particular, contain fragments of nucleic acids, lipids, proteins, and organelles [192]. Notably, apoptotic cells release ATP, which can serve as a signaling molecule by binding to purinergic receptors on cell membranes, activating intracellular signaling pathways, and potentially inflammasome assembly. For instance, ionotropic receptors such as P2XRs are nucleotide-gated ion channels that open upon the binding of ATP. This allows the influx of sodium and Ca^2+^ and the efflux of potassium. Importantly, when intracellular levels of Ca^2+^ increase, it activates p38-MAPK or phospholipase A2 signaling, while P2X_7_R can activate the inflammasome assembly [193]. The interplay between EVs, mtDAMPs, and purinergic receptors may contribute to the induction of inflammation in the heart during CKD, further complicating the cardiorenal connection [194]. Uncontrolled inflammation driven by these mechanisms could contribute to chronic inflammatory diseases [195]. In summary, the activation of TLR9 and the involvement of EVs and mtDAMPs create a complex web of interactions that link renal damage to cardiac inflammation and dysfunction in the context of CKD. Understanding these pathways is crucial for developing targeted interventions to mitigate the CRS observed in these patients.

### 2.7. The Role of Autophagy and Mitophagy in NLRP3 Signaling Pathway in CRS Type 4

Autophagy consists of vesicular sequestration of cellular components, inducing their degradation and further recycling [196]. This process comprises initiation, elongation, fusion, and degradation and is regulated by the phosphoinositide-3 kinase (PI3K) and Unc-51-like kinase (ULK) complexes [197]. The activation of ULK depends on the AMPK protein that phosphorylates and inhibits the mammalian target of Rapamycin complex 1 (mTORC1). PI3K is activated after the autophagic protein Beclin is disassembled from Bcl2, forming the PI3K complex to produce phosphatidyl inositol triphosphate (PI3P).

On the other hand, mitophagy is a specialized form of autophagy that removes damaged mitochondria and is crucial for immune system vigilance and mitochondrial quality control. Mitophagy occurs when the mitochondrial membrane potential (ΔΨ) is disrupted and involves PTEN-induced kinase (PINK) and E3 ubiquitin ligase (Parkin) proteins. Both in autophagy and mitophagy processes, sequestosome p62 proteins (p62) are necessary for degradation because these proteins recognize and ubiquitinate damaged organelle and protein aggregates. Moreover, the microtubule-associated protein 1A/1B-light chain 3 (LC3) is involved in the elongation of autophagosomes [198]. Importantly, the two processes may occur in response to stimuli like hypoxia, ROS, and starvation.

Mitophagy’s role in NLRP3 inflammasome activation is shown by removing autophagy-related proteins, causing the accumulation of damaged mitochondria, and increasing mtDAMPs production [199]. For example, it has been demonstrated that NF-KB restricts NLRP3 inflammasome activation through p62-dependent mitophagy; conversely, the absence of p62 promotes greater mitochondrial damage and increased inflammation [200]. Recently, the role of inflammation and autophagy, specifically mitophagy, has been proposed in kidney diseases, and it has been more studied in acute kidney injury (AKI) models. For instance, in sepsis-induced AKI, the knockout of PINK increased the expression of inflammasome components, such as NLRP3, ASC, and IL-1β [201]. In vitro, the silencing of PINK and Parkin, in contrast-induced AKI, augmented mitochondrial ROS, producing the induction of NLRP3 [202].

In CKD, Zhuang et al. [203] proposed that proteinuria resulted from inflammasome activation, which induced mitochondrial impairment. The authors suggested that the blockage of the NLRP3/caspase-1 pathway might restore mitochondrial damage. Thus, a relationship between mitochondria and inflammasome might be established. Supporting the latter, using the antioxidant targeting mitochondria mitoTEMPO reduced the upregulation of inflammasome components [204]. However, in CRS type 4, the role of mitophagy and inflammasome activation has been poorly explored. An attempt could be made in the hyperuricemic nephropathy model, where the levels of PINK and Parkin are reduced in the kidney, promoting NLRP3 activation and the production of IL-1β, possibly affecting the heart [205]. Interestingly, in the model of CRS type 3 induced by renal ischemia–reperfusion, it was observed that mitophagy was inhibited in cardiomyocytes, promoting inflammation [206]. Thus, the dysregulation of mitophagy might be a factor in activating inflammation through NLRP3 inflammasome.

### 2.8. MAVS and NLRP3-NF-kB Signaling in CRS Type 4

Another role of mitochondria in NLRP3 activation is associated with mitochondrial antiviral proteins (MAVS). MAVS comprises an N-terminal CARD-like domain and a C-terminal transmembrane domain, essential for MAVS signaling. Notably, the transmembrane domain targets MAVS to the mitochondria in the MOM [207]. The latter allows MAVS to participate in the relocalization and association of NLRP3 with ER and mitochondria organelle clusters [137]. This facilitates NLRP3 oligomerization [208,209]. Furthermore, the signaling function of MAVS with mitochondria is essential to enhance downstream factors such as NF-κB and IRFs [210].

Low active caspase-1, IL-1β, and IL-18 levels induce cytokine production, but higher levels of these molecules can induce cell death by apoptosis or pyroptosis. When NLRP3 is activated and associated with MAVS, it leads to pyroptosis [211].

Pyroptosis is a caspase-1-dependent death mediated by the cleavage of gasdermin D by caspase-1 and the subsequent formation of stable pores in the cell membrane [212,213]. The pores formed by gasdermin D proteins promote cell swelling and lytic cell death, releasing cytosolic contents into the extracellular space that act as DAMPs [214]. Also, pyroptosis is regulated through the NLRP3 inflammasome [215]. It could be associated with the release of DAMPs from renal cells, which may activate inflammatory processes in other organs, such as the heart. Accordingly, in uremic cardiomyopathy, increased levels of caspase-1, IL-1β, IL-18, and cleaved gasdermin D p30 protein, the active form of gasdermin D, induced myocardial hypertrophy, interstitial fibrosis, and functional alterations in the heart [216]. Pyroptosis is also associated with renal and cardiovascular diseases [217]. Thus, although NLRP3, MAVS, and pyroptosis are recognized as intrinsic mechanisms underlying the development of cardiovascular alterations during CKD, further research is warranted to comprehensively explore these mechanisms within the context of CRS type 4.

## 3. The Role of the cGAS-STING Pathway in CRS Type 4

### 3.1. The cGAS-STING Pathway

The cGAS-STING pathway plays a pivotal role in mediating inflammation in response to infections, cellular stress, and tissue damage [218]. cGAS activity is triggered by interactions with various ligands, including double-strand DNA (dsDNA), neutrophil DNA–protein complexes, and mtDNA in mammals [218,219]. When cGAS interacts with these ligands, it generates a product known as 2′3′cyclic GMP-AMP [220]. This cyclic GMP-AMP molecule then binds to the STING protein located in the ER membrane [221].

The downstream signaling cascade begins with the translocation of STING from the ER to the Golgi apparatus, facilitated by the ER-to-Golgi transport machinery, specifically the ER–Golgi intermediate compartment (ERGIC) [218,221]. This translocation of STING is a critical step in activating the immune signaling pathway [222,223]. Once in perinuclear compartments, STING forms a complex with TRAF family member-associated NF-κB activator (TANK)-binding kinase (TBK1) [224]. TBK1, in turn, phosphorylates transcription factors, including the interferon regulatory factor 3 (IRF3) and NF-κB [218]. These two factors, IRF3 and the p65 subunit of NF-κB, are the principal downstream effectors of the cGAS-STING pathway [225].

Moreover, it is important to note that the cGAS-STING pathway also functions as a classical PRR, and it can be activated by various myeloid cells and molecular events [217].

### 3.2. The Activation of the cGAS-STING-NF-κB Axis by mtDNA Release in CKD

CKD has been strongly linked to the activation of the cGAS-STING pathway. For instance, in a study conducted by Chung et al. [226], a positive correlation was observed between CKD-induced fibrosis and the expression of cGAS and STING in over 400 kidney tissue samples. Experimental models of diabetic kidney disease and Alport syndrome have shown that the cGAS-STING pathway plays a significant role in the development and progression of glomerular damage by regulating inflammation [227]. Specifically, this pathway is associated with cell damage and chronic inflammation, resulting in the production of inflammatory cytokines and interferons [228].

In CKD, an oxidative stress state is closely related to renal functional and structural alterations, primarily through mitochondrial dysfunction and increased production of ROS [8]. Notably, the plasma of patients receiving platinum-based nephrotoxic anticancer therapy showed elevated levels of mtDNA in plasma, suggesting that STING signaling might be activated through this mechanism [229]. Various mechanisms can lead to the release of nucleic acids in CKD, including increased ROS, apoptosis, mitophagy, and inflammation [230].

The generation of ROS and Ca^2+^ ion accumulation can trigger the opening of the mitochondrial permeability transition pore (mPTP), resulting in the loss of ΔΨ, uncoupling of the ETS, and the release of proapoptotic factors like cytochrome c, which can lead to apoptosis or necrosis. During apoptosis, macropores form in the MOM due to the regulation of BAX and BAK [231,232,233]. These BAX-mediated pores in the MOM allow the inner membrane to herniate, leaking mtDNA and other mitochondrial matrix components in the cytoplasm. The rate of growth of these apoptotic pores and the release of mitochondrial contents depends on the bioavailability of BAX and BAK molecules [234].

In the context of cisplatin-induced nephrotoxicity, it has been suggested that mitochondrial permeabilization induced by BAX pores in the MOM can activate the cGAS-STING pathway, thus triggering inflammation [225]. Small-molecule STING inhibitors, such as H151, have shown promise in ameliorating renal function, kidney morphology, inflammation, and mitochondrial alterations following cisplatin-induced nephrotoxicity [229]. Additionally, activation of the cGAS-STING pathway has been observed in diabetic kidney disease resulting from mitochondrial damage [235]. Zang et al. [236] elegantly demonstrated that the leakage of mtDNA into the cytosol promotes the activation of the cGAS-STING pathway, facilitated by BAX pore formation in podocytes. Furthermore, the transfection of podocytes (MPC5) with cytosolic mtDNA led to cGAS-STING activation and the production of proinflammatory cytokines, dependent on NF-κB p65 and TBK1. The authors also established that BAX macropores are the primary mechanism responsible for mtDNA efflux, causing podocyte injury and cGAS-STING activation.

Additionally, in immune cells, intense STING activation can drive apoptosis, which is induced by the activation of mitochondrial B cell lymphoma 2-homology domain 3 (BH3-only) proteins, ultimately leading to inflammatory signaling [237]. Thus, it can be suggested that mtDNA-cGAS-STING pathway activation and apoptosis of immune cells are critical regulators of inflammation in kidney damage.

Mitophagy is a specialized form of autophagy responsible for selectively removing damaged mitochondria. It is orchestrated by PINK and Parkin [218,238]. Mitophagy is crucial in maintaining cellular health by eliminating compromised mitochondrial components [239]. Consequently, when the process of mitophagy is disrupted or impaired, it contributes to inflammation due to the accumulation of damaged mitochondria [240]. These disruptions lead to an increased production of ROS, triggering the release of mtDNA and the activation of the cGAS-STING pathway [234]. Furthermore, the inadequate packaging of mtDNA, which occurs when mitochondrial transcription factor A (TFAM) is depleted, is a critical regulator for cGAS activity [241]. In line with this, Chung et al. [226] demonstrated that the depletion of TFAM in various experimental models of CKD resulted in the translocation of mtDNA into the cytosol of renal cells. This, in turn, activated the cGAS-STING pathway, thereby promoting renal inflammation and fibrosis.

In normal circumstances, autophagy helps regulate the cGAS-STING pathway by promoting the degradation of STING and terminating its activation signal. This process involves activated STING molecules on the ERGIC binding to LC3 on the autophagy membrane [46]. It is also responsible for the removal of host cytoplasmic DNA through enzymatic degradation within the autolysosome [242]. However, in CKD, there is a disturbance in the autophagic flux [243], suggesting that the STING pathway may become overactivated.

### 3.3. The Activation of the cGAS-STING-NF-κB Axis by mtDNA Release in CRS Type 4

Activation of the immune response in CRS type 4 has been linked to the escape of mtDNA into the cytoplasm, thereby triggering the cGAS-STING pathway [226]. In experimental diabetic cardiomyopathy, the release of mtDNA into the cytosol of heart cells induces inflammation through the cGAS-STING pathway, activating downstream genes, including IRF3, NF-κB, IL-18, and IL-1β [219]. IL-1β, in particular, can potentially disrupt mitochondrial homeostasis by amplifying immune reactions through its activation of cGAS via mtDNA [244,245].

In experimental models of uremic cardiomyopathy, mitochondrial oxidative stress emerges as a consequence of CKD. Oxidative stress triggers the voltage-dependent anion channel (VDAC)-mediated MOM permeabilization, leading to the release of mtDNA and subsequently activating the STING-NF-κB pathway within the heart [41]. DNA fragments released from metabolic organs, originating from the body’s own cells, promote chronic inflammation as they serve as endogenous ligands for the cGAS-STING pathway [227]. Intriguingly, clinical studies have also shown that STING signaling can be activated in patients with metabolic diseases due to the release of mtDNA [246,247].

**Figure 4:**
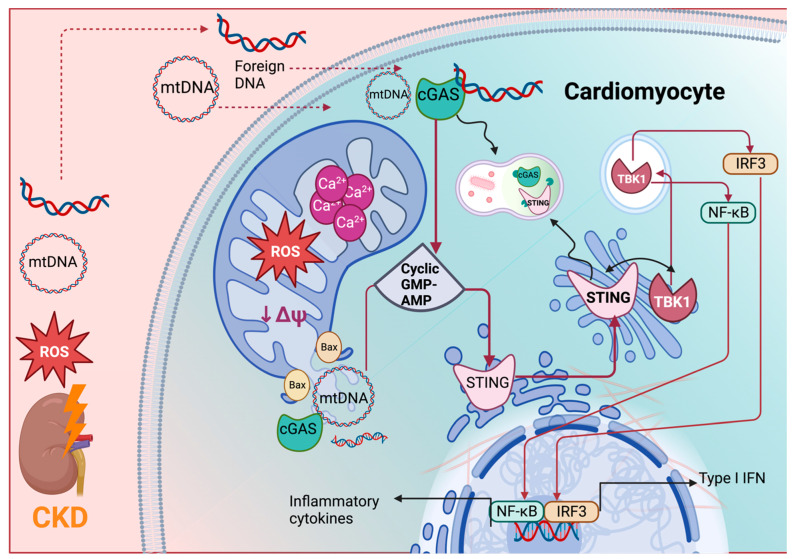
The cyclic guanosine monophosphate (GMP)–adenosine monophosphate (AMP) synthase (cGAS)–stimulator of interferon genes (STING) pathway is activated during cardiorenal syndrome type 4. In chronic kidney disease (CKD), reactive oxygen species (ROS) and other factors cause the release of mitochondrial deoxyribonucleic acid (DNA) fragments that might travel to the cardiomyocytes of the heart. Mitochondrial DNA (mtDNA) or foreign DNA enters the cell and activates cGAS, beginning the signaling cascade. In addition, external perturbations cause damage to cardiac mitochondria, inducing the opening of the mitochondrial outer membrane (MOM) via the pro-apoptotic B-cell lymphoma proteins B-cell lymphoma 2 (BCL-2)-associated X (Bax) and promoting the release of mtDNA. The latter activates cGAS. cGAS activation forms the product 2′3′cyclic GMP-AMP, which induces cyclic GMP-AMP binding to the STING protein in the endoplasmic reticulum (ER) membrane. Then, STING translocates from ER to Golgi, forming a complex with the Tumor necrosis factor receptor (TNFR)-associated factors (TRAF) family member associated NF-κB activator (TANK)-binding kinase (TBK1), delivering TBK1 to endolysosomes. TBK1 phosphorylates the interferon regulatory (IRF3) and nuclear factor-kappa B (NF-κB) in endolysosomes, promoting their translocation to the nucleus to induce the transcription of type I interferon (IFN) and inflammatory cytokines and chemokines, respectively. Finally, c-GAS and STING are degraded via autophagy. ΔΨ: mitochondrial membrane potential. Figure created by using Biorender.com.

Furthermore, experimental studies have confirmed that the release of mtDNA into the cytoplasm of cardiac cells induces inflammation by activating both the cGAS-STING and NF-κB pathways [146,219]. Consequently, the activation of NF-κB in the myocardium through cGAS-STING signaling during CKD is closely associated with the leakage of mtDNA into the cytosol (as illustrated in Figure 4). In addition, Han et al. [44] observed that ornithine decarboxylase–putrescine metabolic flux was transactivated by NF-κB, triggering cardiac hypertrophy in CKD. By contrast, pharmacologic inhibition of STING myocardial mitochondria prevented CKD-associated cardiac hypertrophy. This, in turn, leads to the overproduction of cytokines and chemokines within the heart.

## 4. Chemokines Activation and the Pathophysiology of CRS Type 4

### 4.1. Chemokines Overview

In the context of the heart, inflammation resulting from a uremic state and mitochondrial dysfunction often leads to endothelial dysfunction, oxidative stress, atherosclerosis, vascular calcification, and progressive tissue damage [248,249]. This suggests that the dysregulation of NF-κB via TLRs, NLRP3, and cGAS-STING could serve as a mechanism underlying chronic heart inflammation and the overproduction of chemokines in CRS type 4.

Chemokines are small-molecular-weight chemotactic cytokines [250] that play pivotal roles in directing the migration of neutrophils and monocytes during both acute and chronic inflammation [251]. These chemokines are classified into the following subfamilies, including CXC, CC, XC, and CX3C, based on the position of conserved cysteine residues in their N-terminal domain [252]. The signaling pathways activated by chemokines commence upon their binding to specific chemokine receptors, which are members of the seven-transmembrane G protein-coupled receptor family, a feature unique to vertebrates. It is worth noting that a single chemokine can be recognized by multiple receptors, underscoring their diverse functions in various cell types [69,253].

CC chemokines, characterized by two adjacent cysteine residues, primarily attract monocytes and macrophages through distinct receptors [254]. On the other hand, CXC chemokines feature two cysteine residues separated by a single amino acid (C-X-C) [255,256]. The transcription of certain chemokines is modulated by NF-κB, depending on regulatory elements, including the adjacent activating protein 1 and C/EBP elements. The promoter activation of these chemokines relies on the p65 subunit, which recruits the cAMP-response element binding protein (CREB), an intracellular protein responsible for regulating the expression of multiple genes [257].

Inflammatory chemokines are activated during cellular stress or infection [258]. These specialized chemokines primarily recruit monocytes, leukocytes, and effector T cells [249,259]. Inflammatory processes lead to the upregulation of these chemokines on cell surfaces, facilitating the adhesion of leukocytes to the endothelium [159,259]. However, it is important to note that excessive activation of chemokines can exacerbate damage to the host’s tissues [51]. The following sections will delve into the specific chemokines that play a role in the pathophysiology of CKD and CRS type 4.

### 4.2. The Role of Chemokines and Receptors in the Pathophysiology of CKD

In a healthy kidney, various cell types, including endothelial cells, podocytes, mesangial cells, tubular epithelial cells, and interstitial fibroblasts, typically produce low levels of inflammatory chemokines [260,261]. In patients with CKD, these chemokines are predominately induced by pro-inflammatory cytokines and ROS [262].

The primary role of chemokines in the kidney is to facilitate the recruitment of leukocytes and T cells, which play a central in interstitial fibrosis and the progression of CKD [260,263]. Other factors contributing to chemokine activation in CKD include uremic toxins, cyclic adenosine monophosphate (cAMP), growth factors, lipopolysaccharides, low-density lipoprotein (LDL), IFN-γ, and vasoactive substances [253,262,264]. These factors can further upregulate chemokines by influencing NF-κB and other transcription factors [150]. Consequently, an excess of damaging stimuli in CKD can lead to the overstimulation of chemokines, accelerating disease progression.

#### 4.2.1. Monocyte Chemoattractant Protein-1 (MCP-1)/CCL2 and CCR2 Receptor in CKD

CCL-2 is a well-studied chemokine in cardiac and renal diseases, known for its ability to attract monocytes, T lymphocytes, and natural killer cells [250]. Excessive activation of CCL2 leads to an overwhelming cellular infiltration and prolonged inflammatory response, exacerbating tissue damage and affecting kidney function [265,266]. Upregulation of CCL2 by NF-κB has been linked to tubulointerstitial injury in proteinuric renal disease [267]. Conversely, reducing protein accumulation in renal disease has been shown to decrease CCL2 levels [268].

In advanced CKD, the TGF-β/Smad2,3 pathway activation induces CCL2 expression in renal cells, resulting in a chemotactic effect on macrophages [269]. Likewise, in the UUO model, a well-established model for studying fibrosis in CKD, a wide expression of CCL2 is observed, leading to macrophage infiltration, tubulointerstitial CCL2 expression, leading to macrophage infiltration via a TGF-β/Smad3-dependent signaling pathway [270,271]. Therefore, CCL2 plays a pivotal role in progressive interstitial fibrosis in CKD.

Blocking chemokine activity through neutralizing antibodies, chemokine receptor antagonists, and targeted receptor gene disruption has been shown to prevent glomerular, tubular, and interstitial injury in renal disease models [69,261]. For instance, in a two-month study of acute renal failure in mice with Goodpasture syndrome, CCL2 inhibition using an anti-oligodeoxynucleotide reduced monocyte infiltration and preserved renal function [272] (Table 2).

This experimental model involves glomerulonephritis development due to autoantibody accumulation in the basement membrane [274], eventually leading to renal failure. Similarly, targeted deletions of CCR2, a primary receptor for CCL2, impaired monocyte recruitment to inflammation sites in diabetic nephropathy [256], emphasizing their role in mediating macrophage recruitment. Additionally, disruption of CCL2 or CCR2 helps prevent the excessive production of Th1-type cytokines [258,275], which have immunosuppressive effects by regulating Treg cells [276,277]. Thus, inhibiting CCL2 holds promise as a potential therapy to mitigate excessive macrophage infiltration and fibrotic processes in CKD.

#### 4.2.2. C-C Motif Chemokine 8 (CCL8/MCP-2) in CKD

CCL8 is a CC chemokine that plays a pivotal role in attracting inflammatory monocytes and T lymphocytes in various pathological conditions [278,279]. In advanced CKD and fibrosis-related human glomerulopathies [280], CCL8 levels significantly increase, primarily due to the activation of the TGF-β pathway. Consequently, inhibiting CCL8 has been proposed as a preventive therapy against fibrosis in CKD. In the mice-UUO model, functional blockade of CCL8 with a monoclonal antibody has been shown to prevent fibrosis and apoptosis in renal cells [50] (Table 2). Additionally, inhibiting CCL8 reduces peritoneal inflammation and fibrosis following TGF-β treatment in peritoneal dialysis, underscoring its critical role in promoting inflammation, fibrosis, senescence, and apoptosis [281].

CCL8 has also been associated with early allograft inflammation by promoting the infiltration of CCR8+ T cells. Resident macrophages are the primary sources of CCL8, as blocking CCL8-CCR8 or depleting donor kidney resident macrophages inhibits early allograft immune cell infiltration [282]. Therefore, CCL8’s pathological consequences include fibrosis and early inflammatory processes, possibly driven by transcriptional changes.

#### 4.2.3. Chemokine Interferon-γ-Inducible Protein 10 (IP-10)/Chemokine (C-X-C Motif) Ligand (CXCL)10 in CKD

CXCL10, a member of the CXC chemokine family, exerts its biological functions by binding to the CXCR3 receptor [283]. CXCR3 is expressed in T lymphocytes, natural killer (NK) cells, inflammatory dendritic cells, macrophages, and B cells [284]. CXCL10 is involved in chemotaxis, apoptosis induction, cell growth regulation, and angiostatic effects. It is primarily secreted by leukocytes, activated neutrophils, eosinophils, epithelial cells, and endothelial cells in response to IFN-γ [273]. Once activated, CXCL10 attracts Th1 lymphocytes, monocytes, T cells, and NK cells [273,283]. Interstitial CXCR3 has been implicated in the progressive loss of renal function in human glomerular diseases [260]. While this study did not inhibit CXCR3, reducing CXCL10 expression could potentially decrease the infiltration of immune cells associated with the condition.

In a rat model of renal microvascular endothelial injury, in situ hybridization revealed upregulation of IP-10/CXCL10 in endothelial cells within the tubulointerstitial area. Treatment with a neutralizing anti-CXCL10 antibody resulted in decreased infiltration of tubulointerstitial T cells expressing the CXCR3 receptor and improved renal function [273] (Table 2). The study also unveiled a differential chemokine expression profile by endothelial cells in various renal compartments, underscoring the diverse roles of chemokines in pathological processes.

### 4.3. Chemokines and Receptors in the Pathophysiology of CRS Type 4

Chemokines can also exert inotropic effects, induce cardiomyocyte apoptosis, and contribute to the degradation of the extracellular matrix [51,54]. Notably, the trafficking of leukocytes from the peripheral circulation into the heart involves intricate interactions between soluble mediators, surface molecules on endothelial cells and leukocytes, and the extracellular matrix [260]. Importantly, molecules like mtDAMPs and proinflammatory cytokines such as TNF-α, IL-1β, and IFN-γ can trigger the overexpression of chemokines. This, in turn, induces the production of fibrogenic growth factors and the deposition of extracellular matrix proteins in the cardiac interstitium, leading to an inflammatory response in the heart [162,285].

As a consequence of CKD, inflammatory processes can also manifest in the heart, resulting in significant alterations, including heart failure, coronary artery disease, arrhythmias, and sudden cardiac death. This can ultimately lead to the development of CRS type 4.

#### 4.3.1. Monocyte Chemoattractant Protein-1 (MCP-1)/CCL2 and CCR2 in CRS Type 4

**Figure 5:**
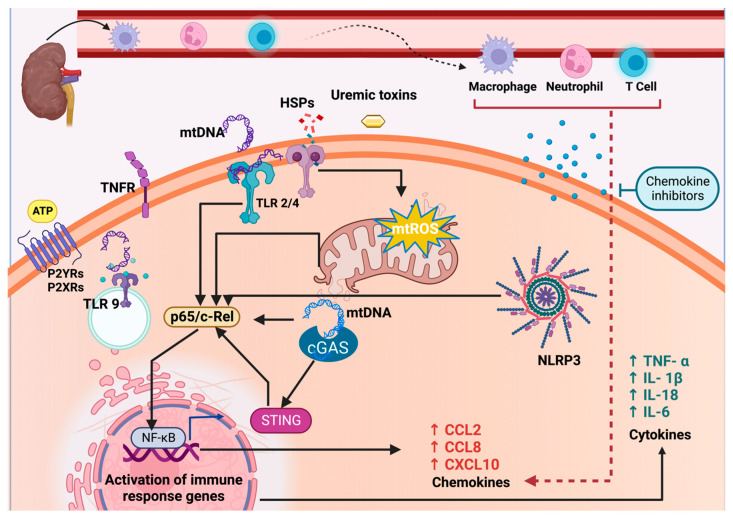
Inflammatory signaling pathways that produce chemokines during cardiorenal syndrome type 4 (CRS type 4). Factors and molecules the kidney releases during chronic kidney disease (CKD) activate inflammation-related signaling pathways. These pathways comprise Toll-like receptors (TLRs) 2, 4, and 9, the stimulator of interferon genes (STING), and NOD-like receptor (NLR) family pyrin domain containing 3 (NLRP3), which mediate the activation of p65/c-Rel, inducing the activation of nuclear factor kappa B (NF-κB) into the nucleus. Moreover, mitochondrial reactive oxygen species (mtROS) stimulates this transcription factor. NF-κB activation induces the production of inflammatory genes like tumor necrosis factor α (TNF-α), proinflammatory interleukins (IL): IL-1β, IL-18, IL-6, and chemokines (CCL2, CCL8, and CXCL10). These chemokines attract inflammatory cells (macrophages, neutrophils, and T cells) to the tissue, aggravating inflammation. Using chemokine inhibitors during CKD might be an excellent strategy to prevent CRS type 4. cGAS: cyclic guanosine monophosphate (cGMP)–adenosine monophosphate (AMP) synthase, mtDNA: mitochondrial deoxyribonucleic acid, HSPs = heat shock proteins, ATP = adenosine triphosphate, TNFR: tumor necrosis factor receptor, P2YRs and P2XRs: purinergic receptor. Symbols: red and blue ↑ mean an increase. Figure created using BioRender.com.

CCL2 is a chemokine that regulates the migration and infiltration of monocytes, macrophages, memory T lymphocytes, and NK cells. In cases of uremia, CCL2 activates chemokine responses through the NF-κB inflammatory signaling in cardiomyocytes [127,286] (Figure 5). Additionally, various uremic toxins elevate CCL2 expression via ROS-induced NF-κB activation in vascular endothelial cells, contributing to cardiovascular changes [287]. While CCL2 is expressed in macrophages, vascular cells, and interstitial fibroblasts, it is also found in cardiomyocytes in rat models of volume-overload congestive heart failure [288].

Increased CCL2 expression is commonly associated with renal and cardiovascular diseases, including heart failure, coronary atherosclerotic heart disease, hypertension, cardiomyopathy, and fibrosis [289]. The latter has been linked to myocardial TGF-β expression, triggering fibrosis [290].

Interestingly, inhibiting CCL2 and its receptor CCR2 has been proposed as a potential therapy in myocardial injury and adverse remodeling [291]. For instance, Kuwahara et al. [292] discovered that CCL2-mediated macrophage aggregation promoted myocardial fibrosis through the TGF-β pathway in a left ventricular pressure overload model. This effect was mitigated by neutralizing CCL2, which reduced macrophage aggregation, inhibited TGF-β induction, curtailed fibroblast proliferation, alleviated diastolic dysfunction, and reduced myocardial fibrosis.

In a myocardial infarction model induced by apolipoprotein E-deficient mice, CCR2 binding to CCL2 led to the trafficking of Ly6C^high^ monocyte subsets, resulting in cardiac remodeling. Silencing CCR2 reduced infarct size, inflammation, and ventricular remodeling. The specific response of CCR2 in the heart was attributed to a reduction in Ly-6C^high^ monocytes [293]. It is important to note that while the association of CCL2 upregulation in CRS type 4 models has not been addressed to date, its role in cardiovascular diseases suggests that it may represent a potential risk factor for developing CRS type 4.

#### 4.3.2. The Role of C-C Motif Chemokine 8 (CCL8/MCP-2) in CRS Type 4

In a previous study, we observed an early transcriptional upregulation of CCL8 during the early stages of CKD in mice, which induced monocyte chemotaxis in the heart [52]. This upregulation correlated with increased infiltration of CD4+T lymphocytes, myeloid cells, and macrophages into the heart. Notably, transient and acute CCL8 inhibition provided protection against several cardiac alterations induced by CKD (Table 3). However, the precise mechanisms underlying the increased levels of inflammatory cytokines and CCL8 in the heart remained unidentified.

It is important to note that the interplay of various factors, including early vascular aging mechanisms, comorbidities, and atherosclerotic processes, influence inflammation during CRS type 4 [294,295,296]. Thus, it is crucial to consider these factors during the initial stages of the disease and its progression, which would be valuable both in experimental models and clinical research.

It is possible that the elevated levels of inflammatory cytokines are initiated by increased TNF-α expression in the heart, which subsequently upregulates CCL8 through TLR4 signaling, recognizing mtDAMPs and inducing NF-κB via the MyD88-dependent pathway [250]. CCL8 has also been implicated in coronary artery diseases, atherosclerosis, and cardiac fibrosis [297,298,299]. Furthermore, its upregulation induces proliferation, migration, cell cycle, changes, and phenotypic alterations in human aortic smooth muscle cells exposed to platelet-derived growth factor BB, serving as a model for atherosclerosis. Silencing CCL8 prevented these changes [300]. Despite these findings, there is limited information in the current literature regarding the role of CCL8 in other forms of CRS. Therefore, further research is required to elucidate the mechanisms associated with CCL8 in CRS. A summary of the therapeutic results promoted by the inhibition of PRRs in CRs type 4 and the associated cytokines and chemokines is presented in Table 4.

#### 4.3.3. IP-10/CXCL10 in CRS Type 4

In a recent study involving two experimental CKD models, CXCL10 chemokine was found to be highly expressed in the heart and associated with monocyte infiltration and local macrophage proliferation [53]. Interestingly, the study identified cardiomyocytes and ventricular fibroblasts as the primary cells responsible for increased CXCL10 expression in the heart. Deletion of the CXCL10 prevented the rise in cardiac F4/80+ macrophages and cardiovascular alterations (Table 3). However, the specific inflammatory pathway associated with CXCL10 upregulation was not investigated.

The activation of NLRP3 inflammasome or cGAS-STING could potentially be linked because the study showed that cardiac fibroblasts, cardiomyocytes, and cardiac endothelial cells responded by expressing profibrotic and pro-inflammatory related genes, such as TGF-β1, IL-1β, and IL-6, which are associated with NF-κB activation (as mentioned above).

Additionally, CXCL10 serves as a chemoattractant for T-lymphocytes in cardiac tissue, endothelial cells, and vascular smooth muscle cells [301]. Elevated CXCL10 levels in CVD contribute to increased cardiac infiltration of proinflammatory Th1 and cytotoxic T cells, resulting in proinflammatory phenotypes [302]. In summary, CXCL10 appears to play a crucial role in CRS development, inhibiting it could be a potential therapeutic target to mitigate this disease.

## 5. Concluding Remarks and Future Perspectives

CKD induces hemodynamic and metabolic changes that lead to mitochondrial damage, causing the release of various components into the peripheral circulation. These mitochondrial components activate inflammatory signaling pathways in organs like the heart, resulting in the upregulation of inflammatory genes, including chemokines and cytokines, further exacerbating damage. Chemokines play a pivotal role in attracting inflammatory cells, thereby intensifying inflammation, and contributing to the development of CRS type 4 development.

While experimental evidence suggests the potential therapeutic benefit of targeting chemokines in CRS type 4, clinical translation may pose challenges due to an incomplete understanding of the mechanisms underlying heart pathophysiology. Therefore, future research should focus on precisely identifying the immunopathogenic mechanisms responsible for cardiac damage and assessing the outcomes of inhibiting specific chemokines. Such studies are essential for the development of novel strategies that target inflammatory and immunopathogenic mechanisms in CRS type 4.

## Figures and Tables

**Table 1:** Classification and characteristics of the five cardiorenal syndrome subtypes.

CRS Subtype	Description	References
Type 1	It develops when there is an acute deterioration of cardiac function due to conditions such as cardiogenic shock, ADHF, cardiac surgery, and acute coronary syndrome leading to AKI (defined by an increase in serum creatinine ≥ 0.3 mg/dL) or renal dysfunction.	[11,12]
Type 2	It characterizes chronic CVD, such as chronic HF, that leads to CKD. CKD increases the frequency of hospitalizations and deaths from pump failure and arrhythmia.	[2,13,14]
Type 3	Describes a sudden worsening of renal function, such as AKI or glomerulonephritis, causing acute cardiac dysfunction (e.g., HF, arrhythmia, or pulmonary edema).	[15,16]
Type 4	It defines CKD as leading to the progression of CVD. CVD may include decreased cardiac function, diastolic dysfunction, ventricular hypertrophy, or increased risk of adverse cardiovascular events due to pressure and fluid overload, representing a risk factor for death.	[17,18]
Type 5	This syndrome appears when an acute or chronic systemic disease such as diabetes mellitus, sepsis, systemic lupus erythematosus, vasculitis, and sarcoidosis, leads to simultaneous cardiac and renal dysfunction.	[11]

Abbreviations: ADHF: acute decompensated heart failure, AKI: acute kidney injury, CKD: chronic kidney disease, CRS: cardiorenal syndrome, CVD: cardiovascular disease, HF: heart failure.

**Table 2:** Chemokines and inhibitory strategies in experimental CKD studies.

Chemokine/ Chemokine Receptor	Inhibitory Strategy	Species (Mice or Rats)	CKD Type	Beneficial Effects	Reference
MCP-1/ CCL2	Blocking of MCP-1 by injecting antisense oligodeoxynucleotide	Rats	Two months model of Goodpasteur syndrome.	↓ MCP-1 mRNA. ↓ Mononuclear cell infiltration. ↓ Monocity/macrophages in the interstitium. ↓ Tubulointerstitial damage.	[272]
CCL8	Anti-CCL8 mAb	Mice	UUO mouse model (14 days). End-stage CKD in the obstructed kidney.	↓ Fibrosis and apoptosis. ↓ E-cadherin and BCL-2. ↓ Fibronectin. ↓ CCR2.	[50]
CXCL10/ IP-10	Inhibition by anti-IP-10/CXCL10 antibody	Rats	Rat model of renal endothelial microvascular injury in CKD.	↓ Tubulointerstitial T cell recruitment. Improved renal function. ↓ Serum creatinine. ↓ BUN.	[273]

Abbreviations: MCP-1: monocyte chemoattractant protein-1, mAb: monoclonal antibody, mRNA: messenger ribonucleic acid, BCL-2: B-cell lymphoma 2, BUN: blood urea nitrogen, CCR2: C-C chemokine receptor 2, CKD: chronic kidney disease, UUO: unilateral ureteral obstruction, CCL2: C-C motif ligand 2, CCL8: C-C motif chemokine 8. Symbols: ↓ decrease.

**Table 3:** Identified chemokines upregulated in the heart during CKD and the benefits of using inhibitory strategies.

Chemokine/ Chemokine Receptor	Inhibitory Strategy	Species (Mice or Rats)	Model	Beneficial Effects	References
CCL8	Anti-CCL8 in early CKD	Mice	Uremic cardiomyopathy induced by 5/6 nephrectomy.	↓ Attenuated infiltration of TCD4+, lymphocytes and macrophages. ↓ Cardiac remodeling. ↓ Inflammation. ↓ Cardiac dysfunction.	[52]
CXCL10	CCR2-/- mice or Anti-CXCL10 antibody	Mice	Uremic cardiomyopathy induced by 5/6 nephrectomy or intraperitoneal folate (240 mg/kg body weight).	↓ Monocyte infiltration in the heart. ↓ Cardiac alterations. ↓ Macrophage local proliferation. ↓ Cardiac hypertrophy. ↓ Cardiac dysfunction.	[53]

Abbreviations: CKD: chronic kidney disease, CCL8: C-C motif chemokine 8, CXCL10: chemokine (C-X-C motif) ligand (CXCL)10. Symbols: ↓ decrease.

**Table 4:** Therapeutic effect of pattern-recognition receptors inhibition and their inflammatory modulation in CRS type 4.

PRR Type	DAMPs or tDAMPs That Activate PRRs	Associated Cytokines/ Chemokines	Therapeutic Effect of the PPR Inhibition in the CRS Type 4	References
NLRP3	↑ ROS Extracellular ATP Cardiolipin EVs Apoptotic bodies	IL-1β, IL-18, TGF-β, TNF-α CCL8, CXCL10	↓ Cardiac dysfunction ↓ Cardiac fibrosis ↓ Cardiac hypertrophy	[121,135,136,142,144,145,146,149,158,192].
TLR2/ TLR4	Cell debris Nucleic acid fragments ↑ Oxidative products ↑ Uremic toxins HSPs	IL-1β, IL-18, IFN-γ, IL-6, TNF-α CCL2, CCL8	↓ Cardiac hypertrophy ↓ BNP levels ↓ α-actin levels	[45,71,131,145,167,168,169,170,171,172,173,174,175,176].
TLR9	mtDNA EVs	IL-1β, IL-18, IL-6 Chemokines?	?	[180,181,182,187,189,193].
cGAS-STING	dsDNA Neutrophil DNA-protein complexes mtDNA	IL-18, IL-1β CCL8, CXCL10	↓ Cardiac hypertrophy	[44,217,218,226,233].

Abbreviations: BNP: brain natriuretic peptide; dsDNA: double-stranded DNA; EVs: extracellular vesicles; HSPs: heat shock proteins; mtDNA: mitochondrial DNA; PRR: pattern-recognition receptor; ROS: reactive oxygen species. Symbols: ↑ increase, ↓ decrease, ? unknown effect.
